# Chlorhexidine Wound Irrigation for the Prevention of Early Surgical Site Infection in Orthopaedic Surgeries in Resource-Limited Settings: A Randomized Controlled Trial

**DOI:** 10.7759/cureus.73235

**Published:** 2024-11-07

**Authors:** Kenneth Ugwoke, Kenechukwu Igbokwe, Obinna M Ayogu, Abdurazaq A Alada

**Affiliations:** 1 Vascular Surgery, United Lincolnshire Hospitals NHS Trust, Lincolnshire, GBR; 2 Trauma and Orthopaedics, Gateshead Health NHS Foundation Trust, Gateshead, GBR; 3 Surgery, National Hospital Abuja, Abuja, NGA

**Keywords:** chlorhexidine gluconate, isotonic saline, low- and middle-income countries (lmics), orthopaedic surgery, resource-limited settings, surgical site infections

## Abstract

Background and objectives

Surgical site infections (SSIs) in orthopaedic surgeries continue to pose significant challenges, leading to increased morbidity and strain on healthcare resources especially in resource-limited countries. Intra-operative wound irrigation is a common practice globally, although evidence supporting this practice is lacking. We aimed to determine the preventative effective of chlorhexidine gluconate (CHG) for prevention of early SSIs in orthopaedic surgeries.

Methods

This is a prospective randomized controlled interventional clinical trial which was designed at National Hospital Abuja, Abuja, Nigeria. A total of 50 patients were enrolled and randomized into two groups: one receiving 0.05% CHG solution and the other receiving isotonic 0.9% saline for wound irrigation. The incidence of early SSIs (within 30 days) postoperatively was the primary outcome measured. Patients and outcome assessors were blinded to intervention allocation.

Results

The results showed no significant difference in the clinical profile of the patients in both groups. There was a similar rate of early SSIs between the CHG (4%) and isotonic saline (4%) groups.

Conclusions

There is no significant difference between CHG and isotonic 0.9% saline in preventing early SSIs in clean orthopaedic implant surgeries. These data will improve care and allow appropriate allocation of scarce resources in resource-limited settings.

## Introduction

Surgical site infection (SSI) remains an undesirable and expensive outcome, accounting for 10-20% of all healthcare-associated infections (HAIs) [1,2]. It is the most frequent type of HAI in low- and middle-income countries (LMICs) and is associated with a high rate of morbidity and mortality postoperatively [1,3]. It impacts patients’ recovery postoperatively, causing pain and discomfort, increasing hospital stay, and increasing the risk of secondary infectious complications, and is implicated in a third of all postoperative deaths [1,4-6]. Several risk factors have been established as determinants for the development of SSIs, such as the degree of contamination of wounds, administration of prophylactic antibiotics, type of operation, ASA score, and patient comorbidities such as diabetes and previous smoking history [5,7,8]. SSIs impact healthcare systems by increasing healthcare costs and resulting in increased readmission rates [1,9].

Originating from exogenous (from the environment of the operating theatres or wards) or from a patient's endogenous flora, a multidisciplinary consensus on superficial and deep SSIs has identified a spectrum of causative pathogens, including *Staphylococcus aureus*, coagulase-negative *Staphylococci*, and gram-negative organisms [10,11]. Given the multifaceted nature of the risk factors involved, prevention demands a holistic “bundle” approach including the use of prophylactic antibiotics, minimally invasive access, wound irrigation, and wound protectors [11-13]. Among these, intra-operative wound irrigation (IOWI) of deep tissues emerges as a critical intra-operative process, aiming to physically cleanse the wound by removing debris and to destroy a broad spectrum of pathogens, thereby potentially reducing bacterial contamination [13].

In this context, the use of isotonic saline has been established in numerous studies, while the 0.05% chlorhexidine gluconate (CHG) solution for IOWI has been proposed as a risk reduction strategy [14]. Various other solutions, including castile soap, antibiotic solutions, and antiseptics such as povidone-iodine or hydrogen peroxide, have been proposed, albeit lacking unanimous support from well-designed clinical trials and healthcare organizations [13,15-16]. The limited availability of these specially treated IOWI options in resource-limited settings like ours has formed the backdrop of this study. Given the paucity of research around these concepts in orthopaedic surgery, especially in resource-limited settings, we believe there is a need to provide evidence for appropriate resource allocation as regards the choice of IOWI: isotonic saline vs CHG in preventing early SSIs in orthopaedic surgery.

## Materials and methods

Design

This trial was a single-centre, randomized clinical trial in which patients undergoing orthopaedic surgeries with implant placements for elective orthopaedic procedures were recruited. The trial is reported in accordance with the Consolidated Standards of Reporting Trials (CONSORT) reporting guidelines [17]. The trial protocol design was approved by the ethics committee of National Hospital Abuja, Abuja, Nigeria (NHA/EC/033/2020). The trial was registered at Nigeria Clinical Trials Registry (nctr.nhrec.net) (Registration number: 2747248). The study was conducted in compliance with the Medical Research Involving Human Subjects Act and the Declaration of Helsinki's principles [18]. Independent monitors assessed the overall performance of study conduct and other issues. This study determined the effectiveness of operative wound irrigation with 0.05% CHG solution in the prevention of early SSIs in orthopaedic implant surgeries at National Hospital Abuja, which is a tertiary referral centre. Patients were randomized to either isotonic saline 0.9% IOWI or 0.05% CHG IOWI in a ratio of 1:1.

Participants

Patients aged 18 to 65 years undergoing open reduction and internal fixation (ORIF) with plates and screws were included in the study. Patients were screened for eligibility at the outpatient clinic. Exclusion criteria were antibiotic treatment at the time of implant placement for a concomitant disease or infection, local and/or systemic symptoms of infection within one week of planned surgery, cancer, uncontrolled diabetes, cardiac failure, a known kidney disease, immunosuppressant use, pregnancy, revision surgeries, and trauma. Patients with open fractures, undergoing surgery with other implants aside plates and screws, with fracture duration > 3 weeks, and with a history of allergy to CHG were not also included. Patient inclusion ran for 12 months from September 2020 to August 2021.

Randomization and blinding

After obtaining written informed consent, eligible patients were randomly assigned to have IOWI with either 0.05% CHG solution or sodium chloride (0.9%) (saline group). These solutions, which are unidentical in appearance, were stored in similar bottles until the time of intra-operative use. The CHG 0.05% w/v (aqueous solution) was locally reconstituted from 5% chlorhexidine at our facility. Participants were randomized into any of the two groups using a computer-generated software program, WinPEPI, which was used to generate a table of numbers (ranging from 1 to 50) in a random sequence. For each case, the first number in the table was used. Numbers 1 to 25 were preassigned to group A (1 L of 0.05% CHG) and numbers 26 to 50 were preassigned to group B (equally gravity-assisted irrigation with only 1 L of normal saline). When the total number of one group was achieved, the remaining participants were assigned to the other group. Patients and outcome assessors were blinded to intervention allocation.

Data collection

Patient and surgical characteristics were collected with a structured proforma. Patient characteristics were sex, age, location of index fracture, SSI following the index fracture, diabetes, alcohol, drug, or nicotine usage, and body mass index (BMI), which is determined by dividing weight in kilograms by height in meters squared. Treatment plans for SSI were recorded and categorized into four groups: surgical debridement, oral antibiotics, intravenous antibiotics, and local wound care without antibiotics.

Sample size calculation and statistical analysis

The sample size was determined using the formulae for comparison groups (n₁ and n₂) and that for sample size when studying proportions with a population of less than 10,000 persons [19,20].

n = 2z^2^pq/d^2^

 and

nf = n / (1 + n/N) 

n = n₁ = n₂ 

where N = total population of patients that had implant orthopaedic surgeries done within one year (yearly average 36), n = minimum sample size of each group where total population, N > 10,000, nf = minimum sample size of each group where total population, N < 10,000, z = standard normal deviation corresponding to 95% confidence level = 1.96, q = 1-p, d = expected minimum difference at significance level of 0.05 = 0.10, and p = prevalence of 9.3% at 5% margin of error and 95% confidence interval [21]. The minimum sample size for each study group was 23 participants. To allow for unexpected data losses, an attrition of 10% was added and the sample size was 50.

The collected data were analysed using SPSS Version 20.0 (IBM Corp., Armonk, NY). Frequency tables were generated for variables of interest, and the appropriate statistical tests (chi-squared test, Student t-test, etc.) were applied to determine the significance and the relationships between the two study groups in line with the objectives of the study. A p-value of 0.05 or less was taken as statistically significant.

Procedure

All surgeries were performed in the trauma or main operating theatres, using the standard aseptic and antiseptic techniques. All members of the operating team (surgeons and perioperative nurse) scrubbed from hands to elbows for a minimum of 5 minutes and gowned and double gloved aseptically. The operating site was prewashed and cleaned with 70% alcohol swab, and 10% povidone iodine-soaked gauze on fresh forceps was used to paint the operating field. Sterile drapes and towel clips were used to isolate the operating field.

Participants received intravenous 1 g of ceftriaxone around 30-60 minutes before incision or 10 minutes prior to inflation of tourniquet. A booster dose of 1 g ceftriaxone was given in procedures with blood loss of up to 1,500 mL or when surgery/procedure extended beyond 3 hours. As per hospital policy, both groups received extended antibiotic prophylaxis with intravenous 1 g ceftriaxone every 12 hours for the next 24 hours.

All surgical wounds were irrigated just before wound closure following implant fixation. In group A patients, 1,000 mL of 0.05% CHG solution was used to irrigate the wound from a sterile kidney dish (gravity flow irrigation) and allowed to stay in the wound for a minimum of 1 minute. In group B patients, wound was only irrigated with 1 L of normal saline from a sterile kidney dish into the wound (gravity flow irrigation) and allowed to stay in the wound for a minimum of 1 minute. Following wound irrigation, routine wound closure followed and dressings were applied as per hospital policy.

Patients were seen routinely by a physician (a non-member of the surgical team), who was blinded to patient treatment group assignment, at the outpatient clinic within two weeks after the surgical procedure. Patients also reported on wound problems in their proforma and were followed up in the initial days by telephone. Signs of SSI (warmth, redness, pain, swelling, wound dehiscence, purulent drainage, or temperature >38°C) were documented on a case report form. Patients were instructed to visit the emergency department or outpatient clinic sooner in case of any signs of SSI. In case of SSI, appropriate treatment was started according to local policy.

Outcomes

Diagnosis of SSIs is based on the presence of one of the following: signs of infection (tenderness, swelling, reddenings and elevated skin temperature), purulent discharge from the incision site or from a drain that is placed through a stab wound into the organ/space, and positive result from an aseptically obtained culture of fluid or tissue from the surgical site [7,8].

The primary outcome was an SSI within 30 days after placement of orthopaedic implants, as defined by the criteria applied by the Centers for Disease Control and Prevention (CDC) [22]. Each SSI was classified as either deep or superficial. A superficial SSI involved only the peri-incisional skin or subcutaneous tissue with at least one of the following: (1) microbiological evidence from an aseptically obtained culture or fluid or tissue from the superficial incision, (2) clinical signs or symptoms of infection such as pain or tenderness, localized swelling, redness, or heat, (3) clinical evidence of drainage from the inicision with or without laboratory confirmation, and (4) planned treatment for superficial SSI by an orthopaedic (trauma) surgeon [22]. Deep SSI involved a suspicion of SSI in deeper tissues with at least one of the following: (1) clinically evident purulent drainage through the incision other than the superficial portions, (2) clinically identified abscess or other evidence of infection found during reoperation or by histopathologic or radiologic examination, (3) clinically evident wound dehiscence or deliberate opening by a surgeon when a patient has either temperature higher than 38°C or localized pain or tenderness, unless site is culture-negative, and (4) planned treatment for a deep SSI by an orthopaedic (trauma) surgeon [22].

## Results

There were a total of 50 patients in this study, with 25 randomized into in each group. The cohort comprised 31 males (62%) and 19 females (38%), with a mean age of 38.4 years. The male-to-female ratio stood at 1.6:1, reflecting a diverse and representative patient population.

Co-morbid conditions

The commonest co-morbid condition in both group A and B patients was controlled hypertensive heart disease, noted in eight (32.0%) patients in group A and six (24.0%) patients in group B (x2 = 0.924, p = 0.630). The summary of co-morbid factors is shown in Table 1.

**Table 1:** Summary of associated co-morbid factors ^f^Fisher’s exact test. *χ*2Chi-squared statistic ASA, American Society of Anesthesiologists

Co-morbid factors	Chlorhexidine (n=25), n (%)	Normal saline (n=25), n (%)	χ^2^	P-value
Controlled diabetes mellitus	3 (12.0)	4 (16.0)	0.166	0.684*^f^*
Smoking	1 (4.0)	2 (8.0)	0.355	0.552^f^
Weight loss	1 (4.0)	0 (0.0)	1.020	0.312*^f^*
Recent surgery in other body region	0 (0.0)	1 (4.0)	1.020	0.312*^f^*
Severe obesity/malnutrition	1 (4.0)	0 (0.0)	1.020	0.312^f^
Controlled hypertensive heart disease	8 (32.0)	6 (24.0)	0.397	0.529
BMI				
Underweight	1 (4.0)	2 (8.0)	0.924	0.630
Normal	5 (20.0)	7 (28.0)		
Overweight	19 (76.0)	16 (64.0)		
ASA				
I	16 (64.0)	20 (80.0)	2.137	0.344
II	8 (32.0)	5 (20.0)		
III	1 (4.0)	0 (0.0)		

Preoperative investigation results

The preoperative mean haemoglobin was 13.1 g/dL, while that for group A was 14.3±8.3 g/dL and that for group B was 11.9±1.8 g/dL, with a p-value of 0.124, which is not significant. The median white blood cell count (WBC) for group A was 7.4±3.2 and that for group B was 8.2±3.0, with a p-value of 0.384, which is not significant. The mean fasting blood sugar was 4.8 mmol/L, while that for group A was 4.5±0.7 and that for group B was 6.4±1.0 (Table 2).

**Table 2:** Summary of pre-operative investigations *t*, t-test statistic; SD, standard deviation

Variables	Chlorhexidine, mean ± SD	Normal saline, mean ± SD	t	p-Value
Hemoglobin (g/dL)	14.3 ± 8.3	11.9 ± 1.8	1.381	0.179
White blood cell count (x10^3^/mm^3^)	7.4 ± 3.2	8.2 ± 3.0	0.965	0.340
Neutrophil (%)	64.4 ± 11.8	65.2 ± 12.9	0.226	0.822
Eosinophil (%)	3.2 ± 2.1	3.4 ± 1.7	0.367	0.715
Lymphocyte (%)	26.1 ± 8.1	25.9 ± 12.7	0.022	0.982
Monocyte (%)	3.6 ± 2.1	4.4 ± 2.1	1.228	0.226
Fasting blood sugar	4.5 ± 0.7	5.4 ± 1.1	2.062	0.081

Comparison of operation data

The commonest anaesthesia was general anaesthesia in 29 (58.0) patients, 11 (44.0) in group A and 18 (72.0) in group B, while the second commonest was combined spinal epidural anaesthesia in 16 patients, 12 (48.0) in group A and 4 (16.0) in group B. In group A, 22 (88.0%) patients were operated on by a consultant, while three (12.0%) patients were operated on by a senior registrar under the supervision of a consultant. In group B, 18 (72.0%) patients were operated on by a consultant, while seven (28.0%) patients were operated on by a senior registrar under supervision of a consultant (Table 3).

**Table 3:** Comparison of operation data SD, standard deviation; SSI, surgical site infection

Variables	Chlorhexidine, mean (SD)	Normal saline, mean (SD)
General anaesthesia	11	18
Spinal anaesthesia	12	4
Lead surgeon		
Consultant	22	18
Senior registrar	3	7
Length of stay	6.1 (3.1)	6.5 (3.2)
Superficial SSI	1	1
Deep SSI	0	0

A total of 50 patients were recruited; two patients developed SSI (4%), with one case of SSI (4%) noted in each of the groups (p =1.00). SSI in group A was in a patient who underwent ORIF with plates and screws for proximal tibia fracture, while that in group was in a patient that underwent ORIF with plates and screws for a distal femoral fracture. Also, the surgery duration in the two cases of SSI was noted to be beyond 120 minutes (140 and 165 minutes, respectively). No adverse events were recorded.

## Discussion

IOWI with antiseptic agents ± isotonic saline is a common practice in our centre. Despite the longstanding practice, its efficacy is yet to be determined. The results from this prospective analysis show that there was no difference in efficacy between CHG and isotonic saline. There was an equal incidence of SSI in both groups, hence suggesting that there may be no difference in the efficacy of IOWI with dilute chlorhexidine solution compared to irrigation with isotonic 0.9% saline. There were also no significant differences in the clinical profile (comorbid status, preoperative investigation results) between the two groups. This study recorded a total of two cases of SSI (4%) in all the patients, with one case of SSI noted in each group (p = 1.00). These results are surprising given the bactericidal and bacteriostatic attributes of CHG in comparison to the non-bactericidal/non-bacteriostatic attributes of isotonic 0.9% saline [23]. Frisch et al. found a decrease in the incidence of SSI with CHG in comparison to control; however, this finding was not significant [24]. Schwechter et al. compared the efficacy of various IOWI solutions to determine their efficacy [25]. On the USA300 subtype methicillin-resistant *Staphylococcus aureus* strain, the fewest colony-forming units were seen after irrigation with normal saline + bacitracin + CHG scrub, but these results are to be treated with caution [25].

Chlorhexidine has a fast onset of action and broad-spectrum antiseptic activity against a wide variety of microorganisms. Bacteria uptake of chlorhexidine is rapid typically working within 20 seconds, resulting in bactericidal effects in high concentrations and bacteriostatic in low concentrations [26,27]. It has also demonstrated unique strong affinity for binding to skin and mucous membranes, which could theoretically enhance its efficacy for SSI prophylaxis [28]. Prior research has demonstrated the cytotoxicity of CHG in human cells with varying results [23,29]. Moreover, 0.9% saline, a mixture of sodium chloride and water, has been regarded as a useful agent for IOWI fluid due to its safety profile and cost-effectiveness compared to other agents [29-31]. It acts mechanically via disruption of haematoma formation, which can serve as a medium for bacterial growth, dilution, and removal of nonviable tissues and foreign bodies while allowing wound bed assessment [32]. The isotonic saline also provides the option of assessing the wound bed during irrigation. Because of its isotonicity and non-toxicity to the normal healing process, it carries little negative effects on the tissue as it carries minimal toxicity.

The use of irrigation solutions presents an additional financial burden for LMICs. They cost more, and staff need protection such as shields and waterproof gowns, which further increases the cost of care. These downsides are in agreement with the NICE (National Institute for Health and Care Excellence) guidelines and the CDC regarding the recommendation of IOWI before wound closure routinely [13,33]. Although the benefits of either solution have been proven in a meta-analysis of IOWI practice in abdominal surgery [34], the benefits in orthopaedic surgery for LMICs are not established. The benefits of either solution was not also proven in the multi-centre FALCON trial conducted in abdominal surgeries in LMICs [35]. Adherence to the existing WHO guidelines has substantial cost implications in resource-limited settings for both providers and patients [36]. Therefore, a desperate need for irrigation solutions after orthopaedic surgeries could be safely achieved with a less expensive alternative such as the isotonic saline.

This study has a number of drawbacks. First, the study's setting in a single centre presents intrinsic limitations that could impact its generalizability. Given the multitude of variables with infection, we see the single-centre design as a potential advantage given surgical variables such as the theatre design among other logistics; in addition, implant vendors, assistant staffing, materials, blood management, the surgical approach, and even postoperative dressings can vary between centres and have profound effects on infection potential. On the other hand, quantifying these is challenging at best. We recognize that this is a small sample size, and our study may be underpowered due to the low prevalence of SSI in our subset. Secondly, this study presents a 30-day assessment of implant using surgeries. Implant infections have been proposed to be expected up till three years postoperatively. However, this study aimed to assess the efficacy of IOWI solutions in early SSI. Finally, a cost analysis was not conducted, and it is important to be mindful of the possible cost increase when using commercially available CHG in comparison to other agents.

## Conclusions

The study found no significant difference in early SSI rates between 0.05% chlorhexidine and normal saline irrigation in clean orthopaedic implant surgeries. The overall incidence of SSI was low, and no statistically significant risk factors were identified. Given the small sample size of our study, we would recommend interpreting the results with caution. Further research is essential to fully understand the long-term implications of these results and determine the most appropriate use of chlorhexidine in orthopaedic surgery.
